# The EAL-domain protein FcsR regulates flagella, chemotaxis and type III secretion system in *Pseudomonas aeruginosa* by a phosphodiesterase independent mechanism

**DOI:** 10.1038/s41598-017-09926-3

**Published:** 2017-08-31

**Authors:** Jessica Rossello, Analía Lima, Magdalena Gil, Jorge Rodríguez Duarte, Agustín Correa, Paulo C. Carvalho, Arlinet Kierbel, Rosario Durán

**Affiliations:** 1 Analytical Biochemistry and Proteomics Unit, Institut Pasteur de Montevideo/Instituto de Investigaciones Biológicas Clemente Estable, Montevideo, Uruguay; 2 Unidad de Proteínas Recombinantes, Institut Pasteur de Montevideo, Montevideo, Uruguay; 3 Laboratory for Proteomics and Protein Engineering, Carlos Chagas Institute, Fiocruz-Paraná, Curitiba, Brazil; 4 Instituto de Investigaciones Biotecnológicas Dr. Rodolfo A. Ugalde (IIB-INTECH), Universidad Nacional de San Martín, Consejo Nacional de Investigaciones Científicas y Técnicas (UNSAM-CONICET), San Martín, Buenos Aires, Argentina; 5 Present Address: Unit of Dynamics of Host-Pathogen Interactions, Institut Pasteur, Paris, France

## Abstract

The second messenger c-di-GMP regulates the switch between motile and sessile bacterial lifestyles. A general feature of c-di-GMP metabolism is the presence of a surprisingly large number of genes coding for diguanylate cyclases and phosphodiesterases, the enzymes responsible for its synthesis and degradation respectively. However, the physiological relevance of this apparent redundancy is not clear, emphasizing the need for investigating the functions of each of these enzymes. Here we focused on the phosphodiesterase PA2133 from *Pseudomonas aeruginosa*, an important opportunistic pathogen. We phenotypically characterized *P*. *aeruginosa* strain K overexpressing PA2133 or its inactive mutant. We showed that biofilm formation and motility are severely impaired by overexpression of PA2133. Our quantitative proteomic approach applied to the membrane and exoprotein fractions revealed that proteins involved in three processes were mostly affected: flagellar motility, type III secretion system and chemotaxis. While inhibition of biofilm formation can be ascribed to the phosphodiesterase activity of PA2133, down-regulation of flagellar, chemotaxis, and type III secretion system proteins is independent of this enzymatic activity. Based on these unexpected effects of PA2133, we propose to rename this gene product FcsR, for **F**lagellar, **c**hemotaxis and type III **s**ecretion system **R**egulator.

## Introduction

*Pseudomonas aeruginosa* is a ubiquitous Gram negative bacterium causative of a major public health problem. This opportunistic pathogen is able to infect immunocompromised individuals making it one of the most common causes of hospital-acquired infections^1^. *P*. *aeruginosa* is also responsible for chronic infections, and is the primary cause of mortality in cystic fibrosis patients^2^. A crucial aspect for host colonization and persistence is the ability of *P*. *aeruginosa* to switch between motile and surface-attached lifestyles^3, 4^.

The cyclic nucleotide bis-(3′-5′)-cyclic dimeric GMP (c-di-GMP) is a global second messenger that has a central role in the regulation of bacterial adhesiveness, controlling both cell–cell and cell–surface interactions. Cellular levels of c-di-GMP are determined by its synthesis and hydrolysis catalyzed by diguanylate cyclases and phosphodiesterases, respectively. The current model links low levels of this second messenger with motile planktonic bacteria, while high c-di-GMP intracellular concentrations are associated with increased biofilm formation and sessile lifestyle^5–7^. To exert its effects, c-di-GMP acts at different levels controlling gene expression through transcription factors and riboswitches, as well as protein activities or protein subcellular localization^8–11^.

Genome sequencing revealed a surprisingly large number of genes encoding proteins with diguanylate cyclase (GGDEF) and phosphodiesterase (EAL and HD-GYP) characteristic domains in different bacteria^12^. However, the physiological relevance of this apparent redundancy in the enzymes controlling c-di-GMP levels is still not clear. The analysis of *P*. *aeruginosa* PAO1 genome revealed the presence of 17 GGDEF domain containing proteins, 5 EAL domain phosphodiesterases, and 16 hybrid proteins carrying both domains^13^. In addition, many of these enzymes present accessory domains, including sensory input and two-component receiver domains, suggesting that c-di-GMP synthesis is regulated by different environmental signals^14, 15^. Most of these enzymes are part of the core genome of *P*. *aeruginosa* and were found in several clinical and environmental isolates using a DNA microarray^13^. A growing body of evidence suggests that specific diguanylate cyclases and phosphodiesterases are linked to different processes, ranging from the control of flagella and pili function, to the regulation of expression of adhesins, exopolysaccharide operons and virulence genes^6, 7, 16–20^. Analysis of the phenotypes related to the overexpression or deletion of each GGDEF and EAL containing gene in *P*. *aeruginosa* suggested that not all the diguanylate cyclases or phosphodiesterases have the same role on biofilm formation and cytotoxicity^13^. These observations points to a c-di-GMP mediated signalling network far more complex than previously thought; thus deserving further investigation of the molecular function(s) of each GGDEF and EAL containing protein.

In this work, we focused on the EAL domain containing phosphodiesterase PA2133. This 285 amino-acid predicted membrane protein (PSORTb V3.0)^21^ is an active enzyme containing a modified EAL domain (ETL)^13^. According to previous reports, overexpression of this enzyme impairs biofilm production and cytotoxicity, two processes relevant for disease development^13^. Here, we phenotypically characterized *P*. *aeruginosa* strain K (PAK) overexpressing PA2133 and showed that biofilm formation and motility are severely impaired. Further investigation of the membrane and exoprotein fractions by quantitative proteomics revealed that proteins involved in three processes are mainly affected, namely flagellar motility, type III secretion system (TTSS) and chemotaxis. While inhibition of biofilm formation can be ascribed to the phosphodiesterase activity of PA2133, other proteomic and phenotypic changes are also observed using an enzymatically inactive mutant. In particular, down-regulation of flagellar, TTSS, and chemotaxis proteins does not rely in phosphodiesterase activity. Thus, we suggest renaming PA2133 FcsR, for **F**lagellar, **c**hemotaxis and type III **s**ecretion **R**egulator.

## Results

### Expression of FcsR increases c-di-GMP phosphodiesterase activity in whole cell extracts

This work characterizes a *P*. *aeruginosa* PAK strain overexpressing the EAL phosphodiesterase FcsR or its inactive single point mutant FcsRE_60_A. It is well known that the substitution of the conserved glutamic acid residue of the EAL signature by alanine abolishes the activity of phosphodiesterases^22^. Both PAK/pJN-FcsR and PAK/pJN-FcsRE_60_A presented the same colony morphology and growth rate as wild-type PAK (Supplementary Figure 1).

To evaluate c-di-GMP hydrolyzing activity, cell extracts of PAK, PAK/pJN-FcsR or PAK/pJN-FcsRE_60_A were incubated with synthetic c-di-GMP at different time points and subjected to HPLC analysis^13^. Peaks with the same retention time as the synthetic standard were collected and analyzed by MALDI-TOF/TOF mass spectrometry to corroborate that corresponded to c-di-GMP (theoretical MH^+^ = 691.06 Da). The identity of the peaks was further certified by comparing their product ion spectra with the c-di-GMP standard and with previously published data^7^ (Supplementary Figure 2a). Chromatographic peak area was used for c-di-GMP quantitation. As expected, PAK/pNJ-FcsR showed the highest phosphodiesterase activity. c-di-GMP levels drop approximately five-fold after 3 h of incubation in protein extracts from this strain, while under the same conditions the levels of this cyclic nucleotide remained practically unchanged in protein extracts from PAK and PAK/pJN-FcsRE_60_A (Supplementary Figure 2b).

### Expression of FcsR generates a biofilm-defective phenotype

**Figure 1:**
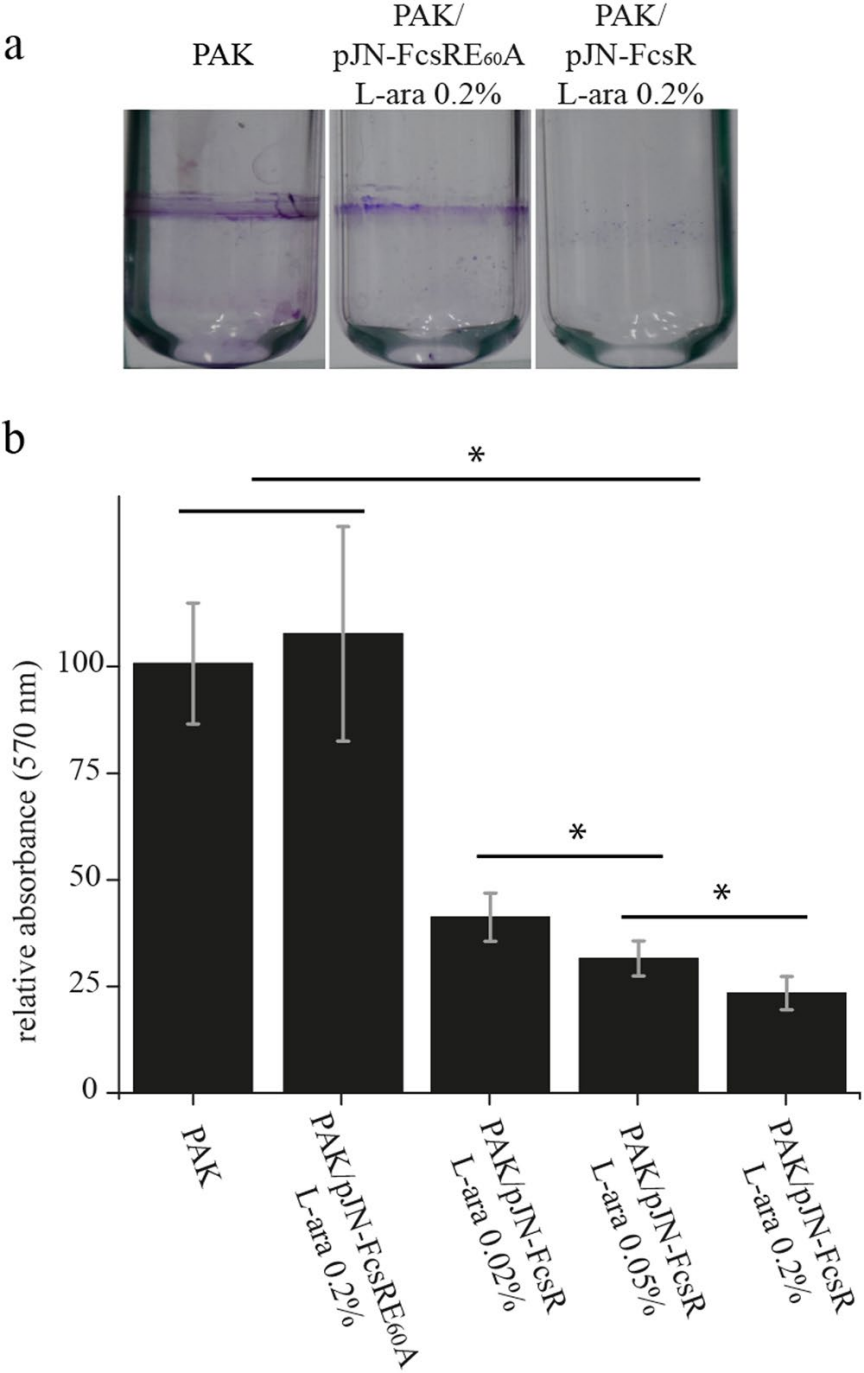
PAK/pJN-FcsR is impaired for biofilm formation. (**a**) Pellicle formation assays. The figure shows the pellicles associated to the glass tubes stained with crystal violet after removal of the liquid culture. This assay was performed for PAK, PAK/pJN-FcsR and PAK/pJN-FcsRE_60_A. The concentrations of L-arabinose used as inducer are indicated. (**b**) Biofilm formation assays. Biofilm formation on solid surface was evaluated using microtiter dish binding assay for PAK, PAK/pJN-FcsR and PAK/pJN-FcsRE_60_A. L-arabinose concentrations used are indicated. Crystal violet staining retained on the biofilms was measured spectrophotometrically at 570 nm for quantification. *Indicates statistically significant difference determined by ANOVA and Tukey’s *post hoc* test (p < 0.05).

Previous work showed that overexpression of FcsR (PA2133) in *P*. *aeruginosa* PA14 strain significantly impairs biofilm formation^13^. To confirm this result in PAK background, we evaluated the ability of PAK/pJN-FcsR and PAK/pJN-FcsRE_60_A to form floating pellicle biofilms at the air/liquid interface, as well as solid-surface associated biofilms. Pellicle formation on glass tubes is shown in Fig. 1a. Wild-type PAK produced a well-developed pellicle; conversely, PAK/pJN-FcsR’s ability to form these multicellular structures was significantly reduced. As expected, the strain overexpressing the inactive phosphodiesterase could form biopellicles to a similar extent as wild-type PAK (Fig. 1a)

In addition, biofilm formation on solid surface was evaluated. As shown in Fig. 1b, induction of FcsR expression with 0.02% of L-arabinose led to a substantial decrease in biofilm formation, with a relative biofilm mass of 41% compared to the control. Moreover, higher L-arabinose concentrations (0.05% and 0.2%) yielded a statistically significant reduction of biofilm mass (32% and 23% respectively) (ANOVA and Tukey’s *post hoc* test, p < 0.05). In line with previous results, expression of the inactive form of FcsR restored biofilm formation to wild-type levels (Fig. 1b).

Overall, our results showed that the expression of the phosphodiesterase FcsR led to defective biofilm formation phenotype that was dependent on its c-di-GMP hydrolyzing activity. To further investigate the function of FcsR at the molecular level, the membrane proteome and exoproteome of PAK, PAK/pJN-FcsR and PAK/pJN-FcsRE_60_A were compared through quantitative approaches.

### Quantitative membrane proteomics reveals that flagella and chemotaxis are altered in PAK/pJN-FcsR and PAK/pJN-FcsRE_60_A

Differentially abundant proteins in membrane enriched fractions of PAK and PAK/pJN-FcsR were shortlisted using a quantitative shotgun approach. In these experiments, the proteins present in biological replicates of each strain were identified using mass spectrometry. Next, the qualitative and quantitative comparisons of these datasets using bioinformatics tools allowed the identification of proteins exclusive to one condition and those whose levels are significantly different between the two conditions. LC-MS/MS analysis resulted in the identification of 651 and 780 proteins in PAK and PAK/pJN-FcsR membrane fractions respectively, with at least two peptides per protein when analysing all replicates per condition (Supplementary Table S1). We verified that 77.5% of the most abundant proteins found in PAK membrane fraction were indeed outer membrane or cytosolic membrane proteins according to *Pseudomonas* genome database^23^ (Supplementary Figure 3).

**Figure 2:**
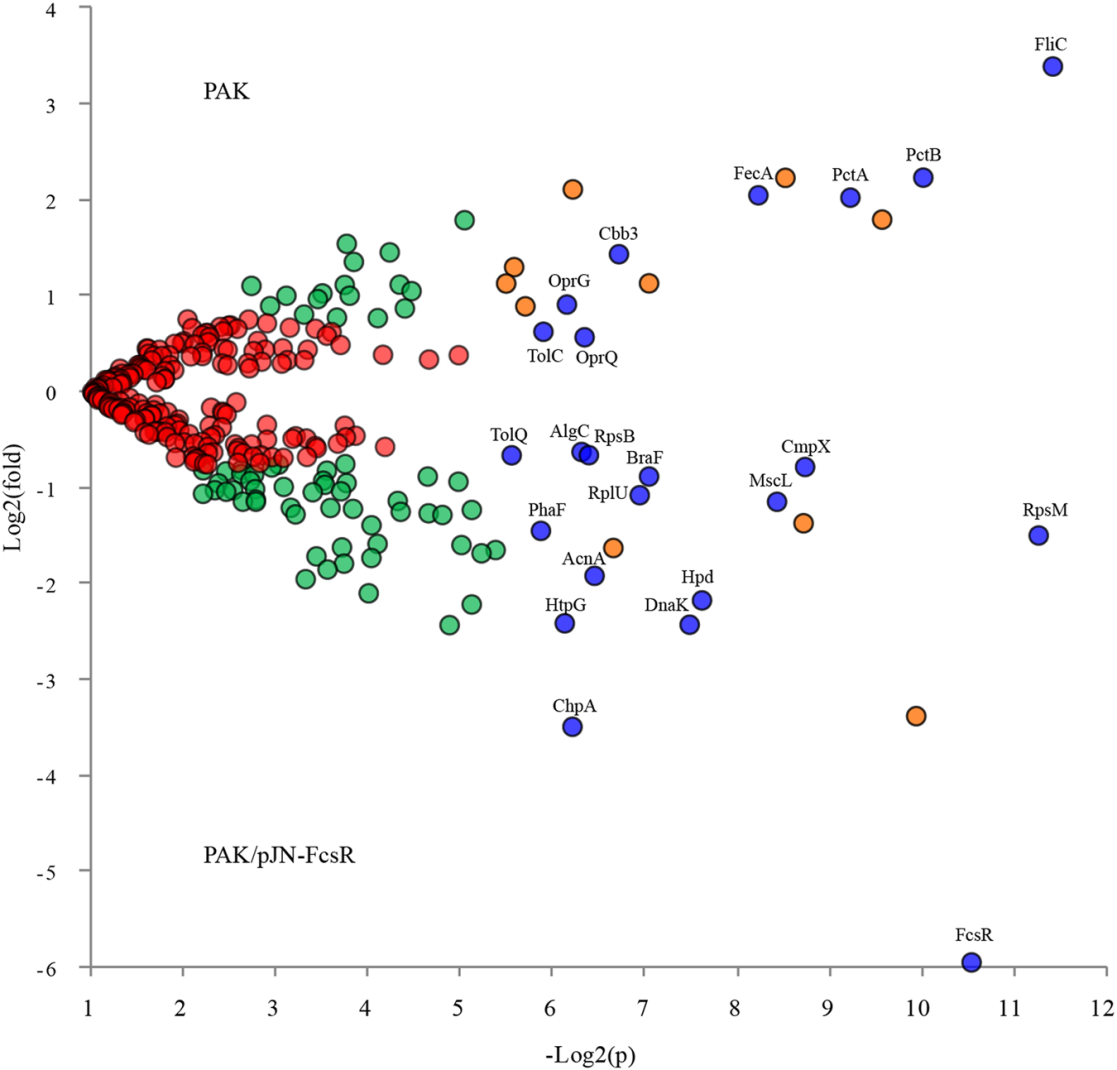
Quantitative analysis of proteins identified in membrane fractions of PAK and PAK/pJN-FcsR using shotgun approach. The figure shows the Volcano plot generated using the PatternLab for Proteomics TFold module. Each dot in the plot represents a protein identified at least in 5 replicates of all conditions, plotted according its p-value (log2 (p)) and fold change (log 2 (fold change)). Red dots represent proteins that do not satisfy neither fold change nor statistic criteria, and thus are considered unchanged between strains. Green dots satisfy fold change criterion but not statistical one. Orange dots correspond to low abundant proteins satisfying both fold change and q value criteria, but due to the low number of spectra they deserved further validation. Finally, blue dots correspond to proteins satisfying all statistical filters and represent the differentially expressed proteins between strains. The identity of each blue dot is shown in the figure (see also Table 2).

Venn diagram module from PatternLab for Proteomics software was used to pinpoint proteins uniquely identified in each strain^24^. Twenty-five proteins were exclusively detected in wild type PAK (in at least 3 of 4 replicates but absent in all PAK/pJN-FcsR replicates); likewise, 9 were unique to PAK/pJN-FcsR (Table 1). Remarkably, the most abundant proteins among those exclusive to PAK were chemotaxis proteins. In fact, 11 out of the 25 PAK unique proteins were methyl-accepting chemotaxis proteins (MCPs). In addition, P- and M-ring proteins of the flagellar basal body were also exclusively detected in wild-type PAK. Among proteins unique to PAK/pJN-FcsR, we identified PelB, PelC and FimL. PelB and PelC are involved in extracellular polysaccharide biosynthesis, while FimL is required for proper twitching motility, biofilm development and TTSS function^25–27^. Next, we compared the proteins present in both strains to identify those that were differentially abundant according to their normalized spectral abundance factors (NSAF). Using the PatternLab’s TFold module statistics and considering five or more replicates in all classes, 23 proteins were shortlisted as differentially abundant (q < 0.05)^28^ (Fig. 2 and Table 2). In line with the above results, the most underrepresented proteins in PAK/pJN-FcsR were A-type flagellin (fold change 10.5) and two methyl-accepting chemotaxis proteins (PctA and PctB) with fold changes of 4.1 and 4.7 respectively. As expected, the phosphodiesterase FcsR was among proteins overrepresented in PAK/pJN-FcsR (fold change 61.6 using 0.2% arabinose). In addition, the His-kinase chemotaxis protein ChpA was also more abundant in PAK/pJN-FcsR membrane enriched fractions (fold change 11.2). These findings were unexpected as it is well known that low c-di-GMP levels are associated with increased flagellar motility and flagellar protein synthesis^7, 29–31^. Interestingly, the analysis of the membrane enriched fraction of PAK/pJN-FcsRE_60_A indicated that flagellar and chemotaxis proteins were also altered when the inactive phosphodiesterase was expressed. We identified 22 proteins in at least 3 PAK replicates that were not detected either in PAK/pJN-FcsR or in PAK/pJN-FcsRE_60_A proteomes, including the previously identified chemotaxis proteins as well as the flagellar P- and M-ring proteins (Supplementary Table S2). Moreover, both FcsR and ChpA were also enriched in membrane fractions of PAK/pJN-FcsRE_60_A, and fold changes were very similar to those observed for PAK/pJN-FcsR (Supplementary Table S3).

**Table 1:** Proteins uniquely identified in the membrane fraction of PAK or PAK/pJN-FcsR.

Description	# replicates	Spectrum count	Gene in PAK	Gene name	Gene in PAO1
**Proteins exclusively detected in membrane fraction of PAK**					
Chemotaxis protein	4	109	PAK_0510		PA4633
Chemotaxis protein	4	107	PAK_02646		PA2654
Chemotaxis transducer	4	84	PAK_02331		PA2867
Chemotaxis protein	4	84	PAK_03742		PA1608
Chemotaxis protein	4	73	PAK_02492		PA2788
Chemotaxis protein	4	62	Y880_0130565	PctC	PA4307
Chemotaxis protein	4	58	PAK_02648		PA2652
Chemotaxis protein	4	48	PAK_02745	CtpH	PA2651
Aerotaxis receptor Aer	4	45	PAK_03791	Aer	PA1561
Flagellar P-ring protein	4	41	PAK_04298	FlgI	PA1084
Membrane protein	4	29	PAK_01662		PA3526
Uncharacterized protein	3	26	PAK_04850		PA4390
Chemotaxis transducer	3	26	PAK_00397	CttP	PA0180
Uncharacterized protein	3	26	PAK_02120		Not found
Flagellar M-ring protein	3	24	PAK_04270	FliF	PA1101
Heme d1 biosynthesis protein NirF	4	22	PAK_00731	NirF	PA0516
Lipoprotein	3	16	PAK_04784		PA4326
Cell division ATP-binding protein FtsE	3	15	PAK_00589	FtsE	PA0374
Acyltransferase	3	14	PAK_00676		PA0461
Chemotaxis protein	3	14	PAK_03895		PA1465
Chemotaxis protein	3	13	PAK_05418		PA4915
Membrane protein	3	12	PAK_04001		PA1365
Histidine kinase	3	12	PAK_04858		PA4398
Uncharacterized protein	3	12	PAK_03420		PA1913
Amino acid ABC transporter substrate-binding protein	3	10	PAK_01924	FecA	PA3268
**Proteins exclusively identified in membrane fraction of PAK/pJN**-**FcsR**					
30 S ribosomal protein S12	4	26	PAK_00903	RpsL	PA4268
Uncharacterized protein	3	32	PAK_02131	PelB	PA3063
Glycosyltransferase family 1	3	14	PAK_02044		Not found
Ferrous iron transporter B	3	11	PAK_03517	FimL	PA1822
Transcriptional regulator	3	13	PAK_01156		PA4021
Heat-shock protein	3	27	PAK_02067	LbpA	PA3126
Appr-1-p processing protein	3	15	PAK_03601		PA1746
Lipoprotein	3	34	PAK_02132	PelC	PA3062
30 S ribosomal protein S17	3	13	PAK_00917	RpsQ	PA4254

**Table 2:** Proteins identified in membrane fractions of PAK and PAK/pJN-FcsR at significantly different levels.

Description	Fold change	*p value*	Gene in PAK	Gene name	Gene in PAO1
**Proteins overrepresented in PAK**					
A-type flagellin	10.5	0.0003	PAK_4280	FliC	PA1092
Chemotaxis protein	4.7	0.0009	PAK_4767	PctB	PA4310
Fe(III) dicitrate transporter	4.1	0.0033	PAK_01276	FecA	PA3901
Chemotaxis protein	4.1	0.0017	Y880_0130550	PctA	PA4309
Cbb3-type cytochrome c oxidase subunit	2.7	0.0095	PAK_3801	Cytochrome oxidase	PA1552
Membrane protein	1.9	0.0141	PAK_1111	OprG	PA4067
Channel protein TolC	1.5	0.0168	PAK_5479	TolC	PA4974
Porin	1.5	0.0123	PAK_2526	OprQ	PA2760
**Proteins overrepresented in PAK/pJN**-**FcsR**					
Cyclic-guanylate-specific phosphodiesterase	61.6	0.0007	PAK_03199	—	PA2133
ChpA protein	11.2	0.0135	PAK_00629	ChpA	PA0413
Chaperone protein DnaK	5.4	0.0056	PAK_5258	DnaK	PA4761
Chaperone protein HtpG	5.3	0.0143	PAK_03754	HtpG	PA1596
4-hydroxyphenylpyruvate dioxygenase	4.5	0.0051	PAK_04521	Hpd	PA0865
Aconitatehydratase	3.8	0.0114	PAK_03790	AcnA	PA1562
30 S ribosomal protein S13	2.8	0.0004	PAK_00930	RpsM	PA4241
Polyhydroxyalkanoate synthesis protein PhaF	2.7	0.0172	PAK_05567	PhaF	PA5060
Large-conductance mechano sensitive channel	2.2	0.0029	PAK_05082	MscL	PA4614
50 S ribosomal protein L21	2.1	0.0081	PAK_05034	RplU	PA4568
High-affinity branched-chain amino acid transport ATP-binding protein BraF	1.8	0.0076	PAK_04311	BraF	PA1071
Cytoplasmic membrane protein	1.7	0.0024	PAK_03572	CmpX	PA1775
Protein tolQ	1.6	0.0214	PAK_04411	TolQ	PA0969
30 S ribosomal protein S2	1.6	0.0120	PAK_01531	RpsB	PA3656
Phosphomannomutase/phosphoglucomutase	1.5	0.0127	PAK_05836	AlgC	PA5322

Altogether, our results indicate that FcsR expression alters the membrane proteome and leads to underrepresentation of chemotaxis and flagellar proteins by a phosphodiesterase independent mechanism.

### Exoproteome analysis reveals that flagella and TTSS are altered in PAK/pJN-FcsR and PAK/pJN-FcsRE_60_A

**Figure 3:**
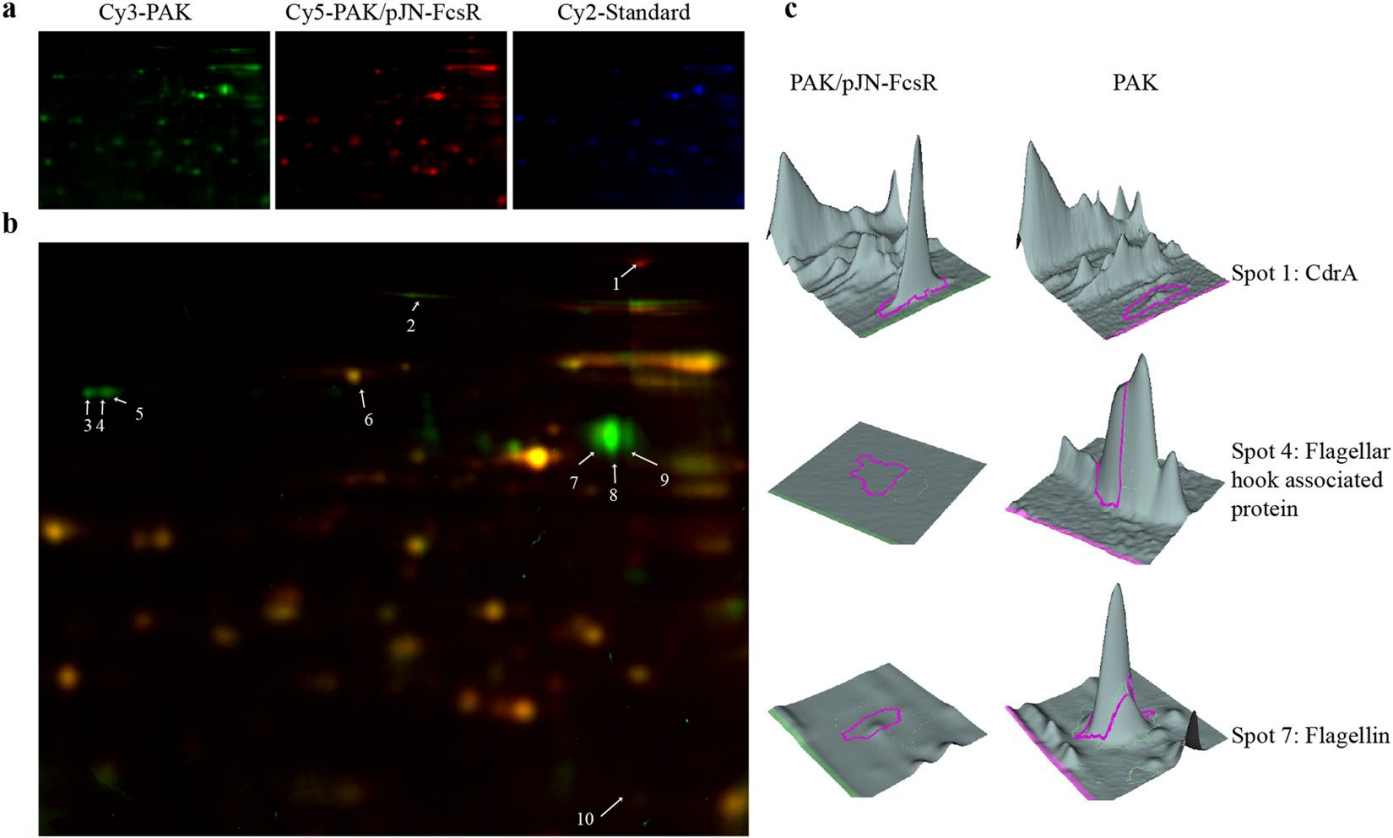
Comparative analysis of exoproteomes of PAK and PAK/pJN-FcsR by 2D DIGE. (**a**) Multiple images for one DIGE gel showing spot profiles for PAK (Cy3 labeled, green), PAK/pJN-FcsR (Cy5 labeled, red) and internal standard (Cy2 labeled, blue). (**b**) Overlay image of PAK (green) and PAK/pJN-FcsR (red) channels. Differential spots between strains (considering all four DIGE gels, p-values ≤ 0.05 and fold change ≥ 25%) are indicated and were further identified by MS analysis. Protein identities, fold changes and *p* values are listed in Supplementary Table S4. (**c**) 3D view of selected spots with significant normalized abundance fold changes: spot 1 CdrA (fold change 4.1) spot 4 flagellar hook-associated protein (fold change 11.2) and spot 7 flagellin (fold change 15.2).

The exoprotein fraction was analyzed with different workflows. Difference gel electrophoresis (DIGE) was used to compare four biological replicates from PAK and PAK/pJN-FcsR. A total of 823 spots were detected in the master gel, and 27 of them were differential between strains (95% significance and considering a fold change ≥ 1.25). 15 and 12 spots from PAK and PAK/pJN-FcsR, respectively, presented significantly increased volumes. Figure 3 shows a representative gel where the 10 spots identified by MS analysis are pointed (Supplementary Table S4). Remarkably, 7 out of 8 spots underrepresented in PAK/pJN-FcsR gels corresponded to proteoforms of flagellar structure proteins (Type A flagellin and A-type flagellar hook-associated protein). These spots presented large differences between strains, with fold changes that ranged from 4.5 to 15.2 (Supplementary Table S4). The analysis also identified two proteins overrepresented in PAK/pJN-FcsR, namely CdrA and OprG.

These results were validated by our shotgun proteomic analysis. Supplementary Table S5 shows the list of proteins identified in each replicate of PAK and PAK/pJN-FcsR. The analysis of proteins unique to each strain (detected in at least 2 of 3 replicates of one class and absent in the other class) revealed 7 proteins exclusive to PAK/pJN-FcsR and 80 proteins unique to PAK (Supplementary Table S6). The proteins unique to PAK were mainly related to flagella and TTSS. A-type flagellar hook-associated protein and flagellar hook protein FlgK, were detected with high number of spectral counts in PAK (total spectra 1152 and 341 respectively) but could not be detected in the exoproteome of PAK/pJN-FcsR. In line with this finding five additional flagellar structure proteins (FlgE, FlgL, FlgG, FliK and FlaG), plus the anti-sigma factor FlgM, were exclusively detected in PAK exoproteome. Our data also indicated that TTSS is altered in PAK/pJN-FcsR. In particular, two proteins of the translocation pore (PopB, PopD), the needle-tip protein PcrV, the TTSS regulator PopN, and the exotoxins ExoS and ExoT were detected in PAK but not in PAK/pJN-FcsR (Supplementary Table S6). Indeed, the translocator protein PcrV and the effector ExoS presented marked inter-strain differences, with high number of spectra in PAK replicates but absent in PAK/pJN-FcsR. Analysis of differentially abundant proteins, performed by spectral counting, further supported our previous shotgun and DIGE results (Supplementary Table S7). A-type flagellin was overrepresented in PAK (71.5 fold change) and CdrA was overrepresented in PAK/pJN-FcsR (79.8 fold change).

Next, exoproteome analysis of PAK/pJN-FcsRE_60_A clearly indicated that the proteomics changes described above were mostly independent of phosphodiesterase activity. As shown in Supplementary Tables S8, 7 flagella proteins (FlgK, FlgM, FlgE, FlgL, FlgG, FliK, and FlaG) as well as the group of TTSS related proteins (PopB, PopD, PopN, ExoS and ExoT), confidently identified in PAK, were not detected either in PAK/pJN-FcsR or in PAK/pJN-FcsRE_60_A. Quantitative analysis also revealed that CdrA is largely enriched in the exoproteome of PAK/pJN-FcsRE_60_A if compared with wild-type PAK (Supplementary Table S3).

Thus, the study of different protein fractions using various proteomic approaches showed, in a consistent manner, that overexpression of phosphodiesterase FcsR or its inactive mutant led to down-regulation of proteins involved in flagellar motility, chemotaxis and TTSS; and up-regulation of CdrA, FimL and ChpA. Some of these unexpected proteomic results were further validated at the functional level.

### Flagellar motility inhibition is independent of FcsR phosphodiesterase activity

**Figure 4:**
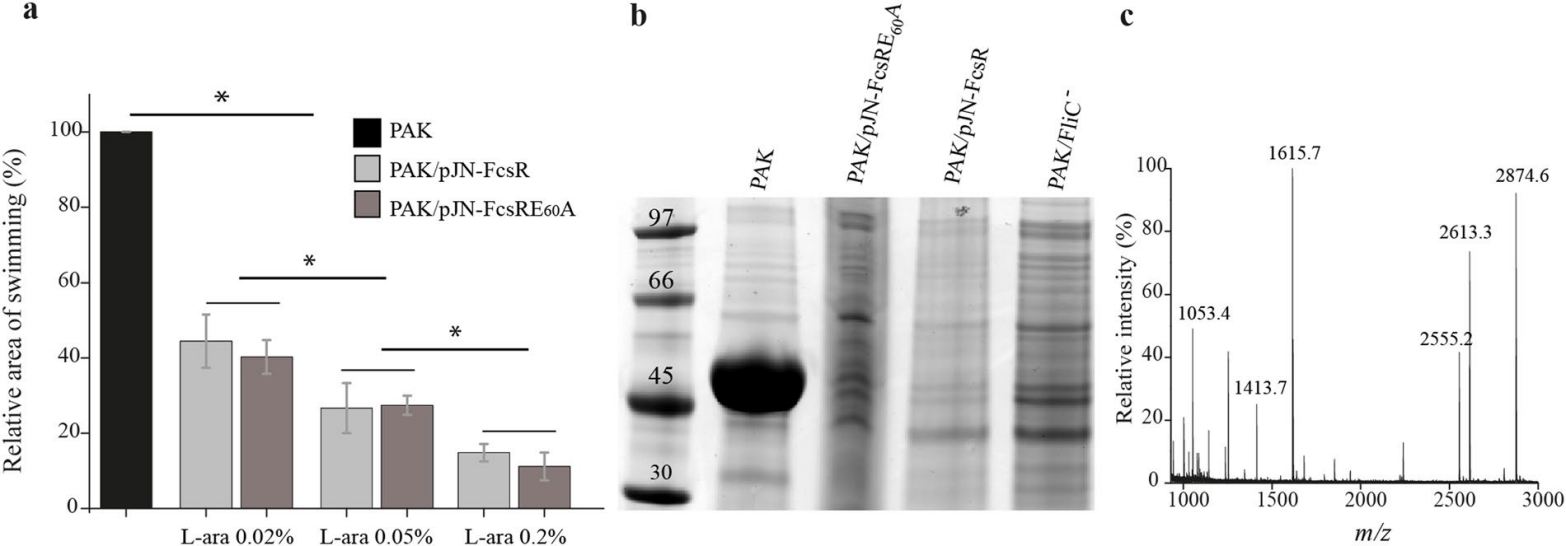
Flagellar motility inhibition is independent of FcsR phosphodiesterase activity. (**a**) Swimming motility assays. Assays were performed in triplicates for PAK, PAK/pJN-FcsR and PAK/pJN-FcsRE_60_A strains. The swimming areas were measured and the relative swimming area (% of wild type PAK) was plotted. The concentrations of L-arabinose used as inducer are indicated. *Indicates statistically significant difference determined by ANOVA and Tukey’s *post hoc* test, p < 0.05. (**b**) Effect of FcsR and FcsRE_60_A on flagella assembly. Flagella isolated from PAK, PAK/pJN-FcsR (0.2% arabinose), PAK/pJN-FcsRE_60_A (0.2% arabinose) and PAK-FliC^-^ strains were analyzed by SDS-PAGE followed by MALDI-TOF/TOF mass spectrometry. The major band of approximately 45 kDa detected in PAK (but absent in all other strains) was unambiguously identified as type A flagellin by mass spectrometry. (**c)** MALDI mass spectrum of tryptic peptides isolated from the 45 kDa gel band in (**b**). The *m/z* values observed can be assigned to FliC sequences (*m/z* 1053.4: sequence 126–135; *m/z* 1413.7: sequence 303–316; *m/z* 1615.7: sequence 2–16; *m/z* 2555.2: sequence 222–247; *m/z* 2613.3: sequence 67–91; *m/z* 2874.6: sequence 368–394).

Flagellar motility was assessed with a standard swimming assay using increasing L-arabinose concentrations. As shown in Fig. 4a, flagellar motility was severely impaired in PAK/pJN-FcsR and the effect was dependent on L-arabinose concentration. Normalized areas of swimming zones for PAK/pJN-FcsR were 45%, 27% and 15% of wild type with 0.02%, 0.05% and 0.2% of L-arabinose respectively (ANOVA and Tukey’s *post hoc* test, p < 0.05). PAK/pJN-FcsR swimming motility in medium without L-arabinose was undistinguishable from wild type (data not shown). Interestingly, PAK/pJN-FcsRE_60_A was also impaired in flagellar motility and this phenotype was more pronounced with increasing inducer concentration (Fig. 4a). Areas of swimming zones were 40%, 27% and 11% of wild type strain using 0.02%; 0.05% and 0.2% of L-arabinose respectively. No significant differences were found between PAK/pJN-FcsR and PAK/pJN-FcsRE_60_A flagellar motility for the same L-arabinose concentration, pointing to a phosphodiesterase activity independent swimming defect.

**Figure 5:**
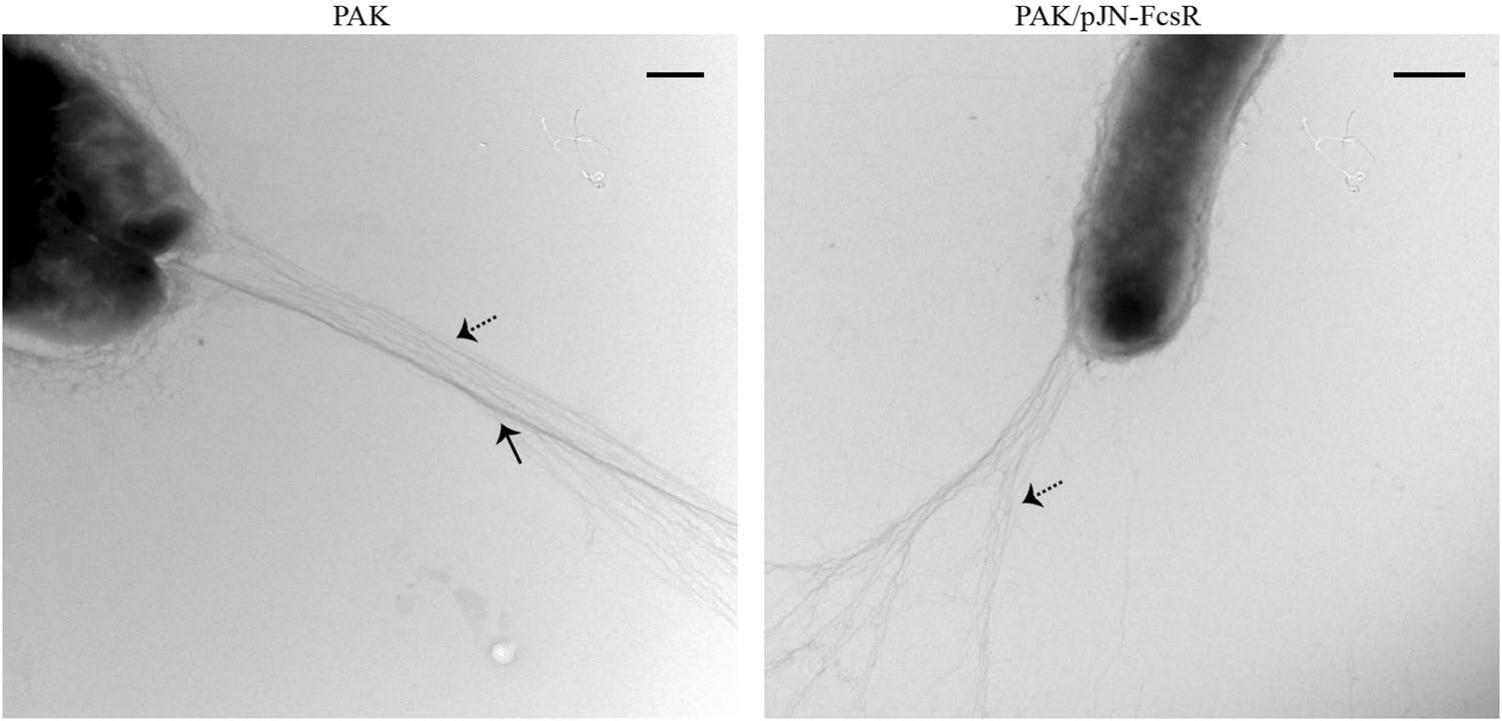
Transmission electron microscopy of PAK and PAK/pJN-FcsR. Electron microscope images showing the presence of flagellum and type IV pili in PAK strain. Only pili were observed in PAK/pJN-FcsR. Flagellum is indicated by a black arrow. Type IV pili are indicated by dashed arrows. Scale bar: 500 nm.

To further investigate the flagellar status of the different strains, flagella isolation was performed. A major gel band of around 45 kDa, identified as type A flagellin (Mascot ion score: 133, sequence coverage: 31%) was detected in PAK but not in PAK/pJN-FcsR, PAK/pJN-FcsRE_60_A or a flagellum negative controls strain (PAK-FliC^−^) (Fig. 4b and c). Moreover, the presence of type IV pili and flagella was clearly seen by transmission electron microscopy in PAK. However, only the pili were present in PAK/pJN-FcsR (Fig. 5 and Supplementary Figure 4). Finally, immunoblotting detected FliC in whole cell extracts of PAK but not in PAK/pJN-FcsR or PAK/pJN-FcsRE_60_A. This result points to the inhibition of biosynthesis of this flagellar structural protein, a process that takes place after assembly of the hook-basal body structure^32^ (Supplementary Figure 5).

Altogether, our proteomic, motility, biochemical and microscopy studies revealed that expression of FcsR led to impairment of flagella motility, and that this effect is independent of FcsR-phosphodiesterase activity.

### TTSS regulation is independent of FcsR phosphodiesterase activity

**Figure 6:**
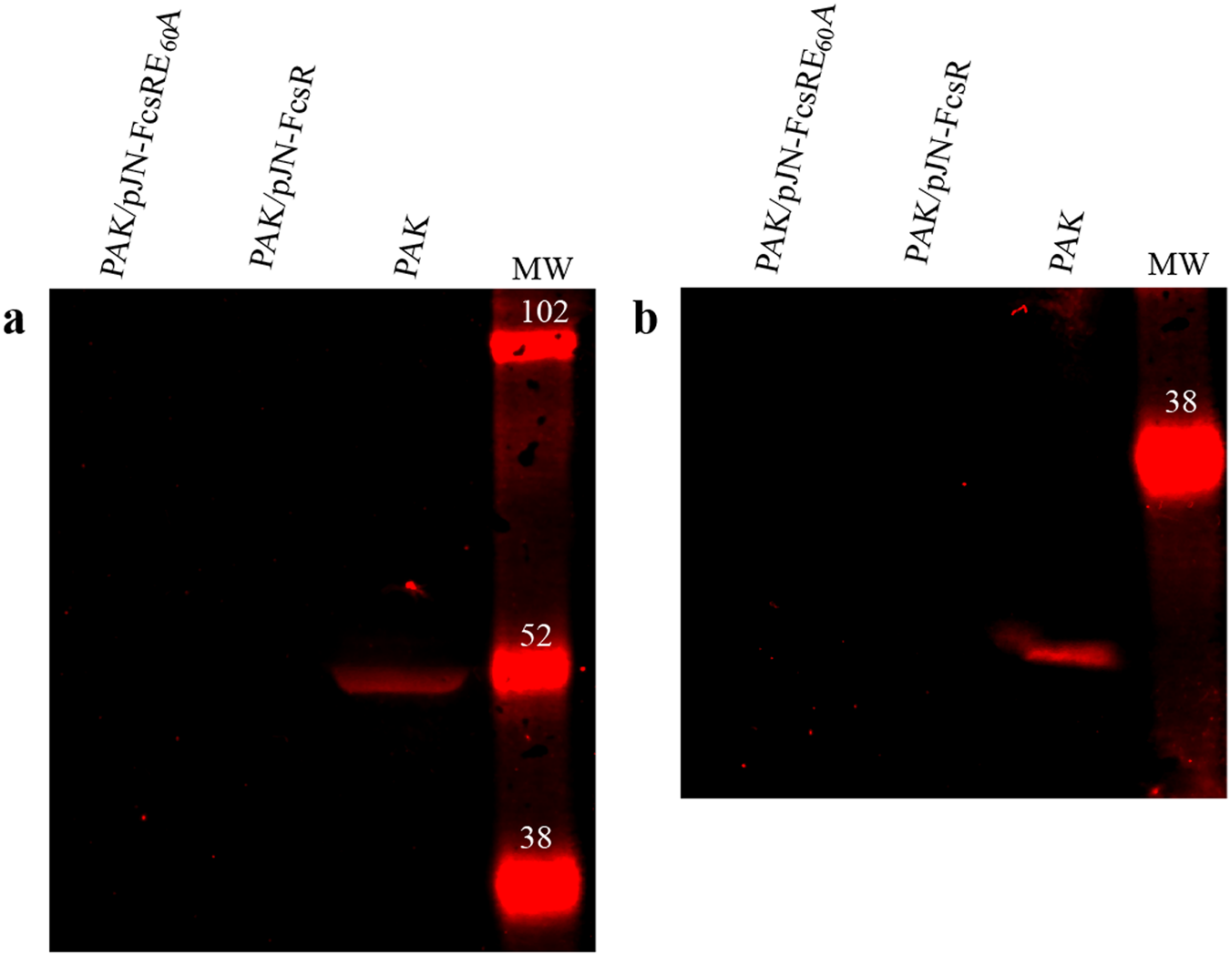
TTSS regulation is independent of FcsR phosphodiesterase activity. Secreted extracts of PAK, PAK/pJN-FcsR and PAK/pJN-FcsRE_60_A grown in Ca^2+^ depleted media were separated on SDS-PAGE and the presence of specific TTSS proteins was detected by Western blot. ExoS and PcrV were sequentially detected in the same blotting membrane. Original images are displayed in Supplementary Figure 7. (**a**) Cropped view of immunoblotting using anti-ExoS antibody. ExoS is only detected in PAK strain (**b**) Cropped view of immunoblotting using anti-PcrV antibody. PcrV is only detected in PAK strain.

Proteomic analysis revealed that FcsR expression led to down-regulation of TTSS proteins. Since those experiments were not conducted under optimum conditions for induction of TTSS, the results were confirmed in Ca^2+^ depleted medium^33^. The presence of PcrV and ExoS in exoproteomes obtained under these experimental conditions was tested by Western blot. In agreement with proteomic results, both proteins were detected in PAK but not in PAK/pJN-FcsR or PAK/pJN-FcsRE_60_A, indicating that the overexpression of FcsR impairs TTSS function and that this effect was independent of phosphodiesterase activity (Fig. 6).

### Flagellar motility phenotype is partially reverted in a ΔChpA strain

Strikingly, two proteins are highly overrepresented in PAK/pJN-FcsR and PAK/pJN-FcsRE_60_A membrane proteome besides FcsR: FimL and ChpA. Interestingly, FimL presents high degree of homology with the N-terminal domain of ChpA^27^. To evaluate if ChpA could mediate some of the phosphodiesterase-independent phenotypes, we transformed PAO1 and PAO1ΔChpA with the pJN-FcsR plasmid. Expression of FcsR in PAO1 background reproduced the motility phenotype observed in PAK, with an average relative swimming area of 22.7% using 0.2% L-arabinose (Supplementary Figure 6). Interestingly, PAO1ΔChpA/pJN-FcsR has an intermediate motility phenotype (swimming area 48.9% of control) (Supplementary Figure 6). Importantly, PAO1ΔChpA has no flagellar motility defect. While we are still far from understanding the mechanism behind this observation, our results suggest that this His-kinase may play a role in the FcsR-phosphodiesterase independent flagellar motility inhibition.

## Discussion

This report examines the role of the EAL-phosphodiesterase PA2133 by overexpressing the active or inactive enzyme in *P*. *aeruginosa* PAK strain. Phenotypic and differential proteomic analyses revealed that this protein regulates four main processes; namely: biofilm formation, flagellar motility, chemotaxis, and TTSS. Our results clearly showed that while biofilm defective phenotype is mediated by FcsR enzymatic activity, the regulation of flagellar motility, chemotaxis, and TTSS does not rely on c-di-GMP hydrolysis.

We showed that PAK/pJN-FcsR is defective in biofilm formation, in agreement with previous reports for strains with low c-di-GMP levels^6, 13, 34–36^. In fact, PAK/pJN-FcsRE_60_A can form biofilm to the same extent as wild type PAK.

On the other hand, two independent proteomic approaches plus phenotypic characterization showed, conclusively, that flagella protein biosynthesis and flagella mediated motility are severely affected in PAK/pJN-FcsR. Overall, 10 flagella structural proteins (that form part of hook, basal body and filament) were underrepresented in the PAK/pJN-FcsR strain when compared to PAK. Differences in abundance of flagella structural proteins were systematically detected using two orthogonal experimental approaches and two different cellular fractions. Particularly A-type flagellin was identified as a differential protein in membrane and exoprotein fractions, while A-type flagellin and type A associated flagellar hook corresponded to 70% of the differential spots in DIGE analysis of exoproteome fraction. Next, motility assays validated proteomics data at the functional level and provided conclusive evidence of swimming motility inhibition. Finally, both electron microscopy and flagella isolation assays indicated that the overexpression of this EAL phosphodiesterase generates mainly an aflagellated strain. The lower abundance of flagella structural proteins in PAK/pJN-FcsR was an unexpected result, as it was previously documented that decreased levels of c-di-GMP correlates with increased flagellin synthesis and flagellar mediated motility^7, 29–31^. Interestingly, we provide strong evidence that the regulation of flagellar motility is not a consequence of low levels of c-di-GMP, as the expression of an inactive mutant produced the same proteomic pattern and swimming defect.

Our results also showed major changes in chemotaxis proteins in PAK/pJN-FcsR strain. *P*. *aeruginosa* genome codifies for 26 methyl accepting chemotaxis receptors and only half of them have been characterized so far^37^. These receptors sense changes in the concentration of attractants or repellents in the environment to generate a behavioral response that directs flagella or pili mediated motility towards or against the chemical gradient. Analysis of membrane proteins indicated that 13 MCP receptors were not detected, or significantly underrepresented, in PAK/pJN-FcsR (corresponding to genes in PAO1: Aer, CtpH, PctC, CttP, PA4633, PA2654, PA2867, PA1608, PA2788, PA2652, PA4915, PctA and PctB), and a similar proteomic profile was obtained for the expression of inactive FcsR. Flagellar motility and chemotaxis are closely related processes that required the ordered expression of a great number of genes. In *P*. *aeruginosa*, the flagella regulon includes 50 genes codifying structural and regulatory flagella proteins, plus chemotaxis system components, that can be classified in four classes according to its temporal expression and the transcriptional regulator involved^38^. Our results show that proteins from Class II to IV were absent in the proteome analysis of PAK/pJN-FcsR and PAK/pJN-FcsRE_60_A.

Finally, we showed that proteins that form part of the TTSS are also underrepresented, and that this secretion system is not induced by calcium depletion in PAK/pJN-FcsR or PAK/pJN-FcsRE_60_A. This multi-protein secretion apparatus is evolutionarily related to flagella and plays key roles in cytotoxicity^39, 40^. These results concur with previous reports showing that a strain overexpressing this phosphodiesterase is defective in TTSS-mediated cytotoxicity^13^. Along with the down-regulation of many flagellar, chemotaxis and TTSS proteins in PAK/pJN-FcsR, we have observed that the levels of several proteins increased in the same strain, including the filamentous haemagglutinin adhesion CdrA. CdrA is a protein involved in adhesion and biofilm formation in *P*. *aeruginosa* whose expression is up-regulated by c-di-GMP, while low levels of this second messenger leads to adhesin release into the extracellular medium and biofilm disaggregation^6, 10, 41–43^.

The coordinated regulation of flagella, CdrA and Pel proteins synthesis through FleQ has been previously reported^10, 41, 44, 45^. FleQ is a c-di-GMP responsive transcription factor that activates the expression of flagella genes and at the same time negatively regulates genes of Pel and Cdr operons. Thus, FleQ is central to the proteomic changes reported here.The expression of FcsR or FcsRE_60_A leaded to increased levels of proteins repressed by FleQ and concomitantly decreased levels of proteins whose expression is activated by FleQ. FleQ expression is regulated by another global transcriptional regulator, Vfr, that directly or indirectly coordinates the expression of more than 200 genes relying on c-AMP dependant and independent mechanisms^46–48^. Among them, Vfr activates TTSS gene expression and represses FleQ transcription^48, 49^. Our proteomics results indicate that the biosynthetic pathways regulated by FleQ and Vfr are altered in PAK/pJN-FcsR and PAK/pJN-FcsR-E_60_A. Indeed, the phenotypes and proteomic profiles described here are reminiscent of the ones observed for Δvfr and ΔfleQ^46, 48, 50^. However, both Vfr (Cyclic AMP receptor protein, protein identifier: S0JAB5) and FleQ (ATPasse AAA with identifier S0IAH2) were detected with low number of spectra in PAK and PAK/pJN-FcsR proteomic analysis, and no statistically significant differences were observed between strains (Supplementary Table S1). The presence of these proteins in the membrane enriched fractions mainly reflects the detection of low abundant cytosolic contaminants using a very sensitive method for protein identification. Under these experimental conditions it is not possible to conclude if the expression levels between strains are different. Thus, the direct interference of FcsR (and FcsRE_60_A) with the expression and/or function of these transcription factors is an interesting hypothesis that needs to be tested.

Together with FcsR, and FcsRE_60_A, a component of the chemotactic signal transduction system, the protein ChpA, was highly enriched in membrane fraction. This protein has a complex domain organization, with six classical and two atypical phosphotransfer domains, in addition to a CheY-like receiver domain^51^. To evaluate if ChpA enrichment in membrane fraction could be part of the molecular mechanism underlying FcsR effects, we express the EAL phosphodiesterase in a strain lacking ChpA. Our results clearly demonstrate that flagellar motility phenotype is at least partially reverted in the absence of ChpA, however, understanding the molecular mechanism behind this observation will require further experimental analysis.

The apparent redundancy of enzymes related to c-di-GMP metabolism in bacterial genomes, together with the observation that strains with similar global c-di-GMP levels can generate distinct phenotypes, have led to the hypothesis that specific interactions between enzymes and their effectors would be responsible of specific phenotypic outputs^13, 52, 53^. Likewise, the analysis of individual mutants of all DGCs and PDEs codified by *P*. *aeruginosa* genome revealed that they display different phenotypes^13^, raising the question about the molecular mechanism underlying its action. The data presented here strongly suggest that FcsR has other roles besides its phosphodiesterase activity, regulating the expression of flagella, chemotaxis and TTSS proteins. Several pieces of evidence support the specific, phosphodiesterase-independent effect of FcsR. First, the swimming motility inhibition inversely correlates with the concentration of L-arabinose used, and we observed a marked motility phenotype even when using low L-arabinose concentrations. Second, the proteomics changes observed are related to specific processes that have been already reported to be jointly regulated. Finally, we showed that the absence of a single regulatory His-kinase, ChpA, partially reverts flagellar motility inhibition. Altogether, the results strongly suggest a specific mechanism of action of FcsR.

Recently phosphodiesterase independent roles were reported for two enzymatically active EAL containing proteins from *E*. *coli*, PdeR and PdeL. The principal role of these trigger phosphodiesterases is to control gene expression through specific interactions with proteins or DNA, while c-di-GMP hydrolysing activity has a secondary role modulating these interactions^54^. PdeR interacts with the MerR-like transcription factor MlrA leading to inhibition of the expression of the biofilm regulator CsgD and its downstream regulated genes involved in curli fibres and biofilm matrix production. Interestingly, a point mutation in the EAL motif renders an inactive enzyme that exerts the same effects on gene expression, but loss its regulation by c-di-GMP^55^. Another example of trigger phosphodiesterase is PdeL that controls transcription by binding directly to DNA^56^. These two enzymes represent a novel type of c-di-GMP-sensing proteins that controls gene expression, and opens the possibility that FcsR may be acting by a similar molecular mechanism, directly or indirectly interfering with master regulators of flagella, chemotaxis and TTSS systems. In the case of FcsR, we could not recognize other protein domains that could be responsible of the phosphodiesterase independent actions. The discovery of the moonlighting phenomenon has largely confronted the one protein-one function dogma. It is now well demonstrated that many bacterial metabolic enzymes have important functions in several processes, including adhesion, virulence and cell signalling, that are independent of its catalytic activity^14, 57^.

Overall our results consistently show that the EAL domain containing protein FcsR participates in the control of processes that are crucial for *P*. *aeruginosa* pathogenesis through a mechanism that does not involve phosphodiesterase activity. It is tempting to speculate that the concept of trigger phosphodiesterases introduced for *E*. *coli* could be a more general feature in c-di-GMP signalling. To unveil the molecular mechanism underlying FcsR’s regulation of motility, chemotaxis and type III secretion system expression further experimentation will be required.

## Methods

### Bacterial strains, plasmids and growth conditions

*Pseudomonas aeruginosa* strains PAK, PAO1, PAO1ΔChpA and its derivatives were grown until stationary phase in Luria–Bertani (LB) broth at 37 °C with shaking, and maintained on LB agar plates. Gentamicin (200 μg/mL) was added to the medium when needed. To evaluate type III secretion system functionality strains were cultured overnight in the presence of 0.2% of L-arabinose, diluted 1/100 in fresh LB containing 0.2% of arabinose and 5 mM EGTA and incubated until late exponential phase. The inactive version of PA2133 used in this work, was generated by site-directed mutagenesis by GenScript® services. Codon GAA, coding for the catalytic glutamic acid residue at position 60 was replaced by GCG which codes for alanine. PAK/pJN-FcsR and PAK/pJN-FcsRE_60_A strains were generated by transforming chemically competent PAK with pJN105 plasmid encoding arabinose-inducible phosphodiesterase PA2133^6^ or its inactive version. The same procedure was used for PAO1 and PAO1ΔChpA strains overexpressing FcsR. L-arabinose was supplemented into the medium at 0.2% (w/v) unless otherwise indicated. PAK-PilA^−^ and PAK-FliC^−^ mutants (kindly provided by J. Engel) were used as type IV pilus and flagellum negative controls respectively.

### Motility assays

Swimming motility was monitored on 0.3% LB agar plates, as previously described^58^. Briefly, bacteria were staked on the top of the agar plates, and grown for 24 h at 37 °C. Plates were imaged and swimming areas were determined using Image J 1.48 s (Wayne Rasband, National Institutes of Health USA). Statistical analysis was performed using ANOVA and Tukey HSD (Honestly Significant Difference) test (p < 0.05).

### Biofilm formation assays

Pellicle formation was assessed by bacteria inoculation into 2 mL of T-broth without salts and statically grown at room temperature. After 72 h cultures were gently removed, and pellicles were stained with crystal violet 0.1%^59^.

Microtiter dish biofilm formation assay was performed as previously described^60^. Biofilm was stained with crystal violet and quantification was performed by measuring absorbance at 570 nm after destaining with 30% acetic acid. Statistical analysis was performed using ANOVA test followed by Tukey test (p < 0.05).

### Electron Microscopy

2 μL of stationary phase cultures were loaded on carbon/formvar grids and negative stained with 2% uranyl acetate. Images were acquired with a Jeol (JEM 10–10) microscope operating at 100 V (Transmission Electron Microscopy Facility, Faculty of Sciences, UdelaR).

### Sample preparation for proteomic analysis

Bacterial cultures grown until stationary phase were centrifuged at 6,000× g. Exoprotein fraction was obtained from supernatants after filtration (0.2 μm pore size) and concentration using centrifugal filter devices (10 kDa cutoff). Membrane enriched fractions were obtained from pellets resuspended in 0.2 M Tris, 1 M sucrose, 1 mM EDTA pH 8, and treated with 1 mg/mL lysozyme. After bacterial osmotic disruption, membranes were resuspended in 40 mM Tris, 4% CHAPS, 10 mM MgCl_2_ pH 8.2, containing 20 μg/mL DNAse and 10 μg/mL RNAse. Samples were centrifuged 30 min at 4 °C and 120,000× g. Pellets containing membrane proteins were resuspended in 0.1 M ice cold sodium carbonate, incubated 1 h at 4 °C and centrifuged 1 h at 120,000× g. Membrane pellets were resuspended in 7 M urea, 2 M thiourea, 4% CHAPS, 1% ASB and 40 mM dithiothreitol solution containing protease inhibitor (Roche).

### 2D-Difference Gel Electrophoresis (DIGE)

Four independent biological replicates of exoproteins of each strain were concentrated using 2-D Clean-Up kit (GE Healthcare) and resuspended in labeling buffer (7 M urea, 2 M thiourea, 4% CHAPS, 30 mM tris pH 8.5). A pool of all samples of each experiment was used as an internal standard. For minimal labeling CyDye DIGE fluors (GE Healthcare) were used. Briefly, 50 μg of each sample were individually labeled with 400 pmol of Cy3 or Cy5. Dye swapping (Cy3 and Cy5) was used to avoid potential bias in labeling efficiencies between samples. The internal standard was labeled with Cy2. Pairs of samples were mixed with an equal amount of internal standard and separated by 2-D electrophoresis. Samples were loaded into 13-cm IPG-strips (pH 3–10) and then were separated by isoelectric focusing (IEF). Disulfide bonds were reduced with dithiothreitol (10 mg/mL) and subsequently alkylated with 25 mg/mL iodoacetamide. The second dimension was performed on hand-cast gels (12% SDS-PAGE). Gels were scanned using a Typhoon^TM^ FLA 9500 scanner (GE Healthcare) using the excitation wavelength and filters settings indicated for each fluorophore. DeCyder 7.2 (GE Healthcare) was used for analysis. Spot co-detection, spot quantification by normalization and ratio calculation were performed using DeCyder Differential In-gel Analysis module (DIA). Gel matching and statistical analyses allowing quantitative comparisons of protein abundance across multiple gels were performed using DeCyder Biological Variation Analysis module (BVA). Student’s t- test was used to assign statistical significance. Protein spots differentially expressed (p ≤ 0.05; normalized abundance ratios fold changes 25%) were further analysed by mass spectrometry (MS).

### Protein identification by MALDI-TOF/TOF

In-gel digestion of selected protein bands or spots was performed overnight at 37 °C by incubation with trypsin (Sequencing grade, Promega). Peptide extraction was performed with 0.1% trifluoroacetic acid (TFA) in 60% acetonitrile as previously described^61^. Samples were vacuum-dried, resuspended in 0.1% TFA, and desalted using C18 OMIX tips (Agilent). Peptides were eluted with matrix solution (α-cyano-4-hydroxycinnamic acid in 60% acetonitrile, 0.1% TFA) directly into the MALDI sample plate. Spectra acquisition was performed on a 4800 MALDI TOF/TOF (Abi Sciex) operating in positive reflector mode. Spectra were externally calibrated using a mixture of peptide standards (Applied Biosystems). MS/MS analysis of selected precursor ions was performed. Database searching (NCBInr 20130706) was performed with Mascot (http://www.matrixscience.com) using the following parameters: unrestricted taxonomy; one trypsin missed cleavage allowed; methionine oxidation as variable modification; carbamidomethylation of cysteine as fixed modification; peptide tolerance of 0.05 Da and a MS/MS tolerance of 0.3 Da. Significant protein scores (p < 0.05) and at least one peptide with significant ions score (p < 0.05) per protein were used as criteria for positive identification.

### LC-MS/MS

Exoproteins and membrane enriched fractions were run on 1 cm long SDS gels (12% acrylamide). In-gel Cys alkylation was performed by incubation with 10 mM dithiothreitol for 1 h at 56 °C followed by incubation with 55 mM iodoacetamide at room temperature for 45 min prior to in-gel digestion. In gel-digestion and peptide extraction was performed as described above. Tryptic peptides were separated using a nano-HPLC (EASY-nLC 1000, Thermo Scientific) coupled to an LTQ Velos mass spectrometer (Thermo Scientific). Peptide mixtures were injected into an Acclaim® PepMap C18 nano-trap column (75 μm × 2 cm, Thermo Scientific) and separated on a 50 μm × 150 mm C18 Easy spray column (PepMap® RSLC, 2 μm, 100 Ǻ) at a flow rate of 250 nL/min. Peptide elution was achieved with a 100 min gradient from 5% to 55% of mobile phase B (A: 0.1% formic acid; B: 0.1% formic acid in acetonitrile). Online MS analysis was carried out in a data dependent mode (full scan followed by MS/MS of the top 10 *m/z* in each segment) using a dynamic exclusion list (exclusion duration 45 s).

### LC-MS/MS data analysis

LC-MS/MS data analysis was performed in accordance to the PatternLab for proteomics 4.0 software (http://www.patternlabforproteomics.org) data analysis protocol^24^.The proteome from *P*. *aeruginosa* strain K was downloaded from UniProt (May 2015) (http://www.uniprot.org). A target-reverse database including the 127 most common contaminants was generated using PatternLab’s database generation tool. Thermo raw file were searched against the database using the integrated Comet^62^ search engine (v. 2015.2) with the following parameters: mass tolerance from the measured precursor *m/z* (ppm): 700; enzyme: trypsin, enzyme specificity: fully-specific, missed cleavages: 1; variable modifications: methionine oxidation; fixed modifications: carbamidomethylation of cysteine. Peptide spectrum matches were filtered using PatternLab’s Search Engine Processor (SEPro) module to achieve a list of identifications with less than 1% of false discovery rate (FDR) at the protein level^63^. Results were post-processed to only accept peptides with six or more residues, proteins with at least two peptide spectrum matches. These last filters led to an FDR at the protein level, to be lower than 1% for all search results. PatternLab’s Approximately-area proportional Venn Diagram module was used for pinpointing proteins exclusively identified in one biological condition. For the enriched membrane protein sample, the analysis only considered proteins present in at least three of four replicates of each biological condition but absent in all replicates of the other condition. Likewise, for exoproteins, only those found in at least two of the three replicates in one biological condition but in none of the other. PatternLab’s TFold module was used for pinpointing proteins found in both conditions but having a statistically differential abundance according their spectral counts^28^. TFold module relies on the Benjamini-Hochberg theoretical FDR estimator to maximize the number of identifications that satisfy both a fold-change cutoff that varies with the t-test p-value as a power law and a stringency criterion that aims to filter out lowly abundant proteins that could produce false positives. Proteins present in five replicates in all conditions for membrane proteins or four replicates in all conditions for exoproteins were considered. Only proteins satisfying fold change and p-value criteria were considered as differentially expressed.

To allow comparison between PAK proteins and the reference strain PAO1, the protein blast tool of *Pseudomonas* genome database was used^23^.

### Phosphodiesterase activity assays

Phosphodiesterase activity assays were performed as previously described^13^. Briefly, 25 mL of *P*. *aeruginosa* late-exponential-phase cultures were centrifuged and pellets washed and resuspended in buffer containing 50 mM Tris pH 8, 10 mM MgCl_2_, 250 mM NaCl, 5 mM mercaptoethanol, 1 μM PMSF and complete EDTA-free protease inhibitor (Roche). Cells were lysed by sonication and c-di-GMP standard (Kerafast) was added to the cell lysates of the different strains under investigation (PAK, PAK/pJN-FcsR and PAK/pJN-FcsRE_60_A) to a final concentration of 6 μM. c-di-GMP hydrolysis was followed at different time points using reversed-phase HPLC coupled to MALDI TOF/TOF analysis. Samples were evaporated to dryness using a Speed Vac and resuspended in 200 μL of miliQ water. HPLC analysis was performed by injecting 50 μL of sample in an Agilent 1200 HPLC system fitted with a Hypersyl Gold aQ column (150 × 4.6 mm, Thermo) using a 2 min isocratic step at 1% B followed by a 20 min linear gradient up to 20% of B at a flow rate of 1 mL/min (A: 0.1% TFA; B: 0.1% TFA in methanol). Detection was performed at 280 nm. Collected samples were analyzed on a MALDI-TOF/TOF Instrument (4800, ABi Sciex) in reflector and MS/MS mode (matrix solution: α-cyano-4-hydroxycinnamic acid in 60% acetonitrile, 0.1% TFA).

### Flagella isolation

Flagella isolation was performed according to previous reports^64^. Briefly, *P*. *aeruginosa* cultures were centrifuged; pellets were resuspended in PBS and blended for 1 min at 4 °C. After centrifugation, the supernatants were recovered and centrifuged at 40,000× g for 3 h. The pellet containing flagella was resuspended in 150 μL of PBS, separated in 12% SDS-PAGE gels and the main bands were processed and analysed for MALDI-TOF/TOF as described before.

### Western Blot

For ExoS and PcrV detection, cultures grown in the presence of EGTA as described before were centrifuged and supernatants were concentrated using Amicon centrifuge-filters. Exoproteins of PAK, PAK/pJN-FcsR and PAK/pJN-FcsRE_60_A strains were separated in SDS PAGE gels, and transferred into low fluorescence PVDF membranes before incubation with 1:2,000 dilution of anti ExoS rabbit polyclonal antibody^65^ (kindly provided by J. Shouguang) or 1:500 dilution of anti PcrV rabbit polyclonal antibody^66^ (kindly provided by A. Rietsch). For FliC detection, whole cell extracts were separated in SDS PAGE gels. Proteins were transferred to PVDF membranes, and incubated with 1:20,000 dilution of anti-flagellin polyclonal antibody^67^ (kindly provided by D. Wozniak). Goat anti rabbit antibody coupled to Cy5 (GE Healthcare) was used as secondary antibody. Detection was performed using a Typhoon FLA 9500 (GE, Healthcare).

### Data availability

Proteomics data generated in the present work have been deposited to the ProteomeXchange Consortium (http://proteomecentral.proteomexchange.org) via the PRIDE partner repository^68^ with the dataset identifier PXD004365.

## Electronic supplementary material

Supplementary Information
