# Supplementary material for: The EAL-domain protein FcsR regulates flagella, chemotaxis and type III secretion system in Pseudomonas aeruginosa by a phosphodiesterase independent mechanism
Source: Sci Rep. 2017 Aug 31;7:10281. doi: 10.1038/s41598-017-09926-3 (PMC5579053; doi:10.1038/s41598-017-09926-3)
Supplement: Supplementary file 1 — Supplementary Information [file 41598_2017_9926_MOESM1_ESM.pdf]

Supplementary Information

The EAL-domain protein FcsR regulates flagella, chemotaxis and type III secretion system in *Pseudomonas aeruginosa* by a phosphodiesterase independent mechanism.

Jessica Rossello, Analía Lima, Magdalena Gil, Jorge Rodríguez Duarte, Agustín Correa, Paulo C. Carvalho, Arlinet Kierbel and Rosario Durán

Supplementary information includes:

- **Supplementary Figure 1. Overexpression of FcsR or FcsRE$_{60}$A does not affect growth rate**
- **Supplementary Figure 2. Overexpressing FcsR but not its inactive mutant increases phosphodiesterase activity of cell extracts**
- **Supplementary Figure 3. Subcellular localization of most abundant proteins identified in membrane enriched and exoprotein fractions.**
- **Supplementary Figure 4. Transmission electron microscopy of PAK strains lacking flagella or pili**
- **Supplementary Figure 5. FliC detection by Western blot in whole-cell protein extracts**
- **Supplementary Figure 6. Flagellar motility phenotype is partially reverted in a ΔChpA strain**
- **Supplementary Figure 7. TTSS regulation is independent of FcsR phosphodiesterase activity-Original images of Figure 6**

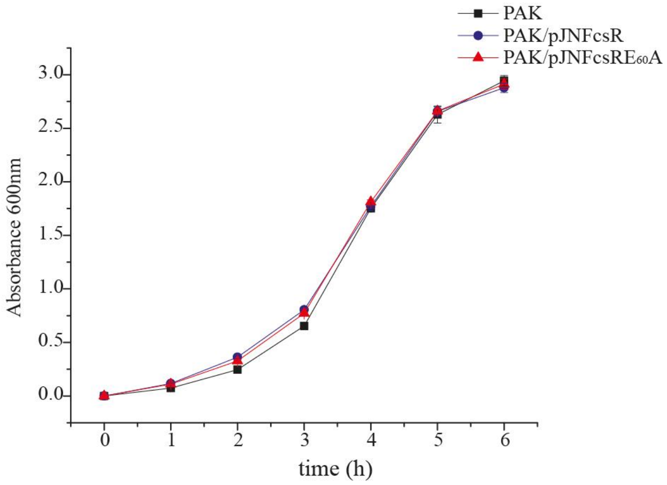

Supplementary Figure 1. Overexpression of FcsR or FcsRE$_{60}$A does not affect growth rate

PAK, PAK/pJN-FcsR or PAK/pJN-FcsRE$_{60}$A overnight cultures containing 0.2% of arabinose were diluted 1/100 in fresh LB broth (with arabinose and gentamicin) and growth was monitored spectrophotometrically. The plot shows the Absorbance at 600 nm at different time points for each strain. The data represent the average of the measurements carried out in triplicate; error bars represent the standard deviation.

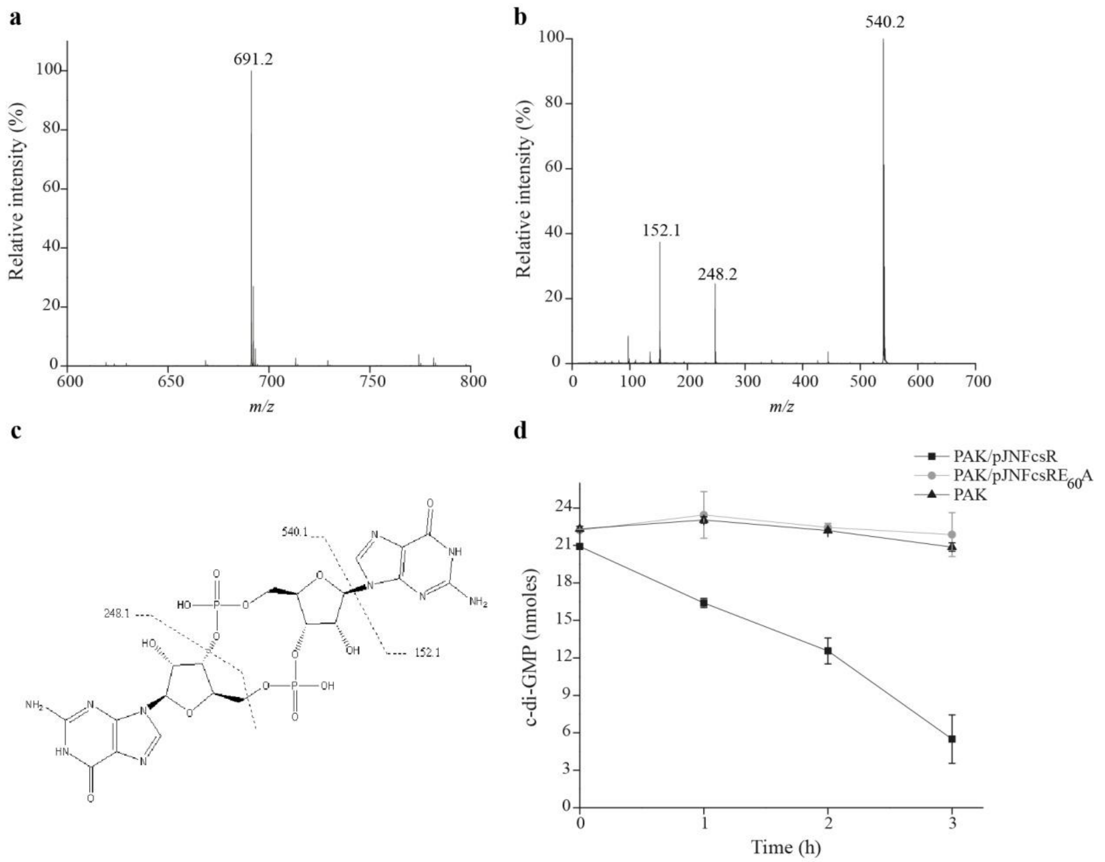

Supplementary Figure 2. Overexpressing FcsR but not its inactive mutant increases phosphodiesterase activity of cell extracts

a. Representative mass spectrum of c-di-GMP recovered from reaction mixtures (protein extracts incubated with synthetic c-di-GMP) after HPLC separation. Chromatographic peaks with the same retention time of the c-di- GMP standard were collected and analyzed by MALDI-TOF MS in reflector mode (matrix solution: α-cyano-4-hydroxycinnamic acid in 60% acetonitrile, 0.1% TFA). A signal of *m/z* 691.2 is observed as expected for the monocharged ion of c-di-GMP. **b.** MS/MS spectrum of the ion of *m/z* value 691.2. **c.** Structure of c-di-GMP showing the main product ions shown in MS/MS spectrum and its corresponding *m/z* values. **d**. Hydrolysis of synthetic c-di-GMP by total cell extracts of PAK, PAK/pJN-FcsR or PAK/pJN-FcsRE$_{60}$A. The amount of c-di-GMP at different time points was determined using a standard curve of synthetic c-di-GMP and considering the area of HPLC peaks. Phosphodiesterase activity measurements were performed in duplicates, error bars indicate standard deviation.

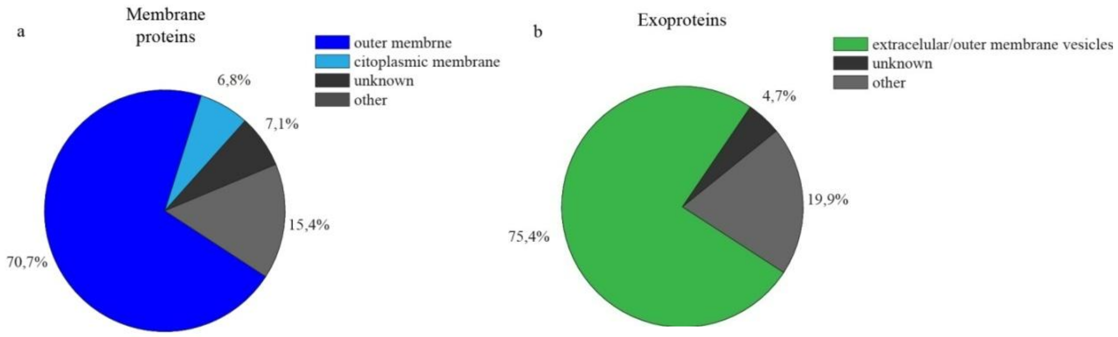

Supplementary Figure 3. Subcellular localization of most abundant proteins identified in membrane enriched and exoprotein fractions

Proteins with 90 or more spectral counts were considered for the analysis, comprising 16058 spectra out of a total of 27384 assigned spectra in membrane enriched fraction and 11445 spectra out of a total of 15842 assigned spectra in exoproteome of WT strain respectively. Subcellular locations were assigned according to *Pseudomonas* database.

a. Subcellular localization of proteins identified in membrane enriched fraction: 70.7% of most abundant proteins correspond to outer membrane localization, 6.8% to cytoplasmic membrane, 7.1% correspond to proteins with unknown localization, and 15.4% comprises proteins that have a different subcellular localization.

b. Subcellular localization of proteins identified in exoproteome: 75.4% of most abundant proteins correspond to extracellular proteins or proteins that have been detected in outer membrane vesicles (green), 4.7% represent proteins with unknown subcelular localization, and 19.9% correspond to proteins with a different subcellular localization.

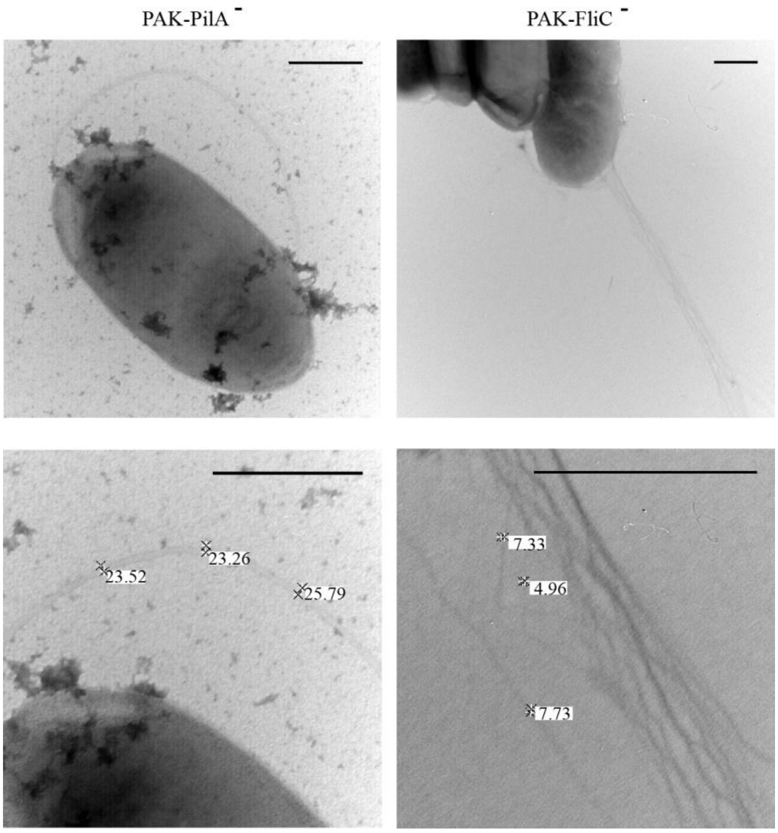

Supplementary Figure 4. Transmission electron microscopy of PAK strains lacking flagella or pili

Electron microscopy showing the lack of flagellum and type IV pili in PAK-FliC^{-} and PAK-PilA^{-} strains respectively. Size measurements of the main structures observed in PAK-FliC^{-} and PAK-PilA^{-} supports the identity of each motility appendix. Scale bar: 500 nm.

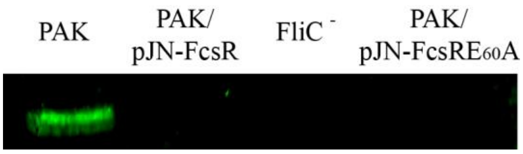

Supplementary Figure 5. FliC detection by Western blot in whole-cell protein extracts

Whole protein extracts of PAK, PAK/pJN-FcsR, PAK/pJN-FcsRE_{60}A and PAK-FliC$^-$ were separated on SDS-PAGE. Proteins were transferred to PVDF membranes, and incubated an anti-flagellin polyclonal antibody (kindly provided by D. Wozniak) followed by the secondary antibody (goat anti rabbit coupled to Cy5). Detection was performed using a Typhoon FLA 9500 (GE, Healthcare).

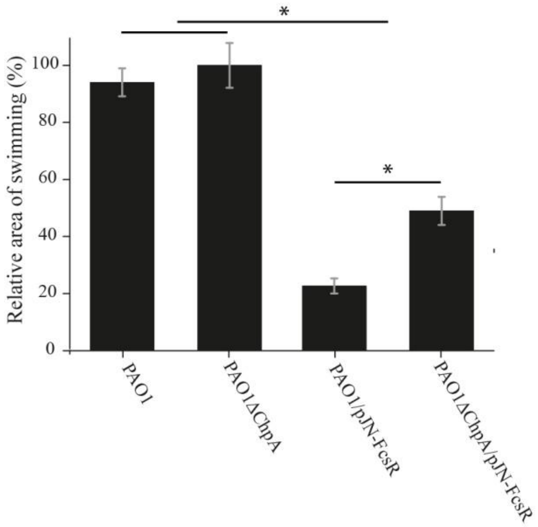

Supplementary Figure 6. Flagellar motility phenotype is partially reverted in a ΔChpA strain.

Quantitation of swimming areas using 0.2% L-arabinose was performed by triplicate as described in methods section for each of the strains under investigation. Swimming areas were measured using Image J and the relative swimming area was plotted. * indicates statistically significant difference determined by ANOVA and Tukey's *post hoc* test, $p < 0.05$.

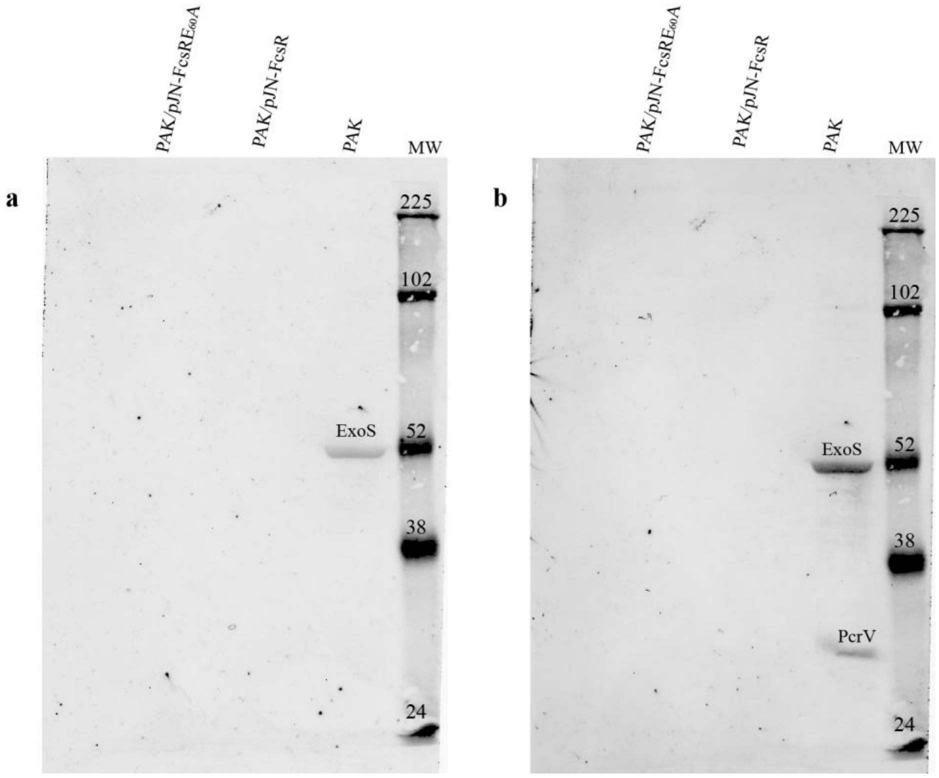

Supplementary Figure 7. TTSS regulation is independent of FcsR phosphodiesterase activity.

Original images of immunoblottings showed in Figure 6. ExoS and PcrV were sequentially detected in the same blotting membrane. **a** ExoS detection using rabbit anti-ExoS. **b** Subsequent PcrV detection using rabbit anti-PcrV. Goat anti rabbit antibody coupled to Cy5 was used as secondary antibody.
